# The Biobanking Analysis Resource Catalogue (BARCdb): a new research tool for the analysis of biobank samples

**DOI:** 10.1093/nar/gku1008

**Published:** 2014-10-21

**Authors:** Joakim Galli, Johan Oelrich, Michael J. Taussig, Ulrika Andreasson, Eva Ortega-Paino, Ulf Landegren

**Affiliations:** 1 Department of Immunology, Genetics and Pathology, Science for Life Laboratory, Uppsala University, SE-751 08 Uppsala, Sweden; 2 Protein Technology Group, The Babraham Institute, Cambridge CB22 3AT, UK; 3 Department of Immunotechnology, Lund University, Medicon Village, SE-223 81 Lund, Sweden; 4 CREATE Health, Lund University Medicon Village, SE-223 81 Lund, Sweden

## Abstract

We report the development of a new database of technology services and products for analysis of biobank samples in biomedical research. BARCdb, the Biobanking Analysis Resource Catalogue, is a freely available web resource, listing expertise and molecular resource capabilities of research centres and biotechnology companies. The database is designed for researchers who require information on how to make best use of valuable biospecimens from biobanks and other sample collections, focusing on the choice of analytical techniques and the demands they make on the type of samples, pre-analytical sample preparation and amounts needed. BARCdb has been developed as part of the Swedish biobanking infrastructure (BBMRI.se), but now welcomes submissions from service providers throughout Europe. BARCdb can help match resource providers with potential users, stimulating transnational collaborations and ensuring compatibility of results from different labs. It can promote a more optimal use of European resources in general, both with respect to standard and more experimental technologies, as well as for valuable biobank samples. This article describes how information on service and reagent providers of relevant technologies is made available on BARCdb, and how this resource may contribute to strengthening biomedical research in academia and in the biotechnology and pharmaceutical industries.

## INTRODUCTION

Biobanks are recognised as invaluable resources for clinical research and are being increasingly used for basic medical research, biomarker discovery, personalised medicine and drug development—indeed wherever sets of well annotated human samples are required. Biobanking activities have increased considerably during the recent decade (1,2). In Europe, initiatives such as BBMRI (the Biobanking and Biomolecular Resources Infrastructure, http://www.bbmri.eu), as well as corresponding national initiatives in Scandinavia and throughout Europe, have sought to coordinate the availability of samples in biobanks (3). Furthermore, these initiatives encompass not only the storage, handling and pre-analytical aspects, as in the U.S. guidelines Biospecimen Reporting for Improved Study Quality (4), but they also provide up-to-date information about methods (‘biomolecular resources’) which can be applied to them. Indeed, biobanks, molecular technologies and reagents for molecular analyses represent a trinity of crucial resources: for optimal function it is necessary to achieve excellence in all three areas in order to take full advantage of opportunities for studies of patient sample collections. The recent rapid development of high throughput techniques for molecular analysis, including genomics (Single Nucleotide Polymorphism (SNP) typing and exome, whole genome and epigenome sequencing), transcriptomics, proteomics, metabolomics and imaging techniques for tissues and cells, has vastly expanded opportunities to collect large amounts of data that offer valuable insights into the aetiology and progress of diseases, as well as enabling identification of biomarkers for disease stratification, prediction and early diagnosis. The central contribution of biobank samples for genetic studies is evident in, e.g. sequencing surveys of cancer genomes (5) and genome-wide association studies (6), while in the protein field there is a particular demand for sensitive and multiplex methods for determination of biomarkers in patient tissue and plasma samples (7), especially in longitudinal studies, and for autoantibody detection in patient cohorts (8).

It is therefore of particular importance to ensure that researchers have broad access to state of the art—and in particular to beyond state of the art—techniques and reagents, in order to ensure that the maximal potential of biobank samples is realised. This efficient connection between techniques, samples and medical questions is also critically important for innovators of molecular techniques. However, it is often difficult to identify suitable technology providers to undertake studies with the required level of throughput and standardisation. In response to this need, we have designed an online database, termed BARCdb (Biobanking Analysis Resource Catalogue), as a freely available resource of information to facilitate optimal use of samples stored in biobanks. BARCdb provides up-to-date information on technologies and reagents for analysis of biobank samples, together with listings of service providers and relevant organisations, together with details of services offered, location and contact information. Initially focused on facilities in the Nordic region as part of the Swedish BBMRI network (http://www.bbmri.se), BARCdb is now being extended to the rest of Europe.

## COMPUTATIONAL DESIGN

BARCdb has been built using the MVC (Model, View, Controller) design pattern, with these three aspects implemented as separate entities. The purpose is to facilitate maintenance and further development as well as testing. The system is implemented in the C# programming language and uses Microsoft ASP.NET, Entity Framework and ASP.NET. MVC.

ASP.NET is a large collection of web-related libraries; Entity Framework helps with the database design and the mapping of database entries to types used by the BARCdb application; ASP.NET MVC is an implementation of the MVC design pattern in ASP.NET. The front end of the system is enhanced with JavaScript and CSS. To render BARCdb easier to use, some pages have been built using suggestions from the SPA (Single Page Application) pattern. An example is the front page where the layout is altered depending on what the user does.

## DATABASE AND SEARCH FUNCTIONS

**Figure 1.**
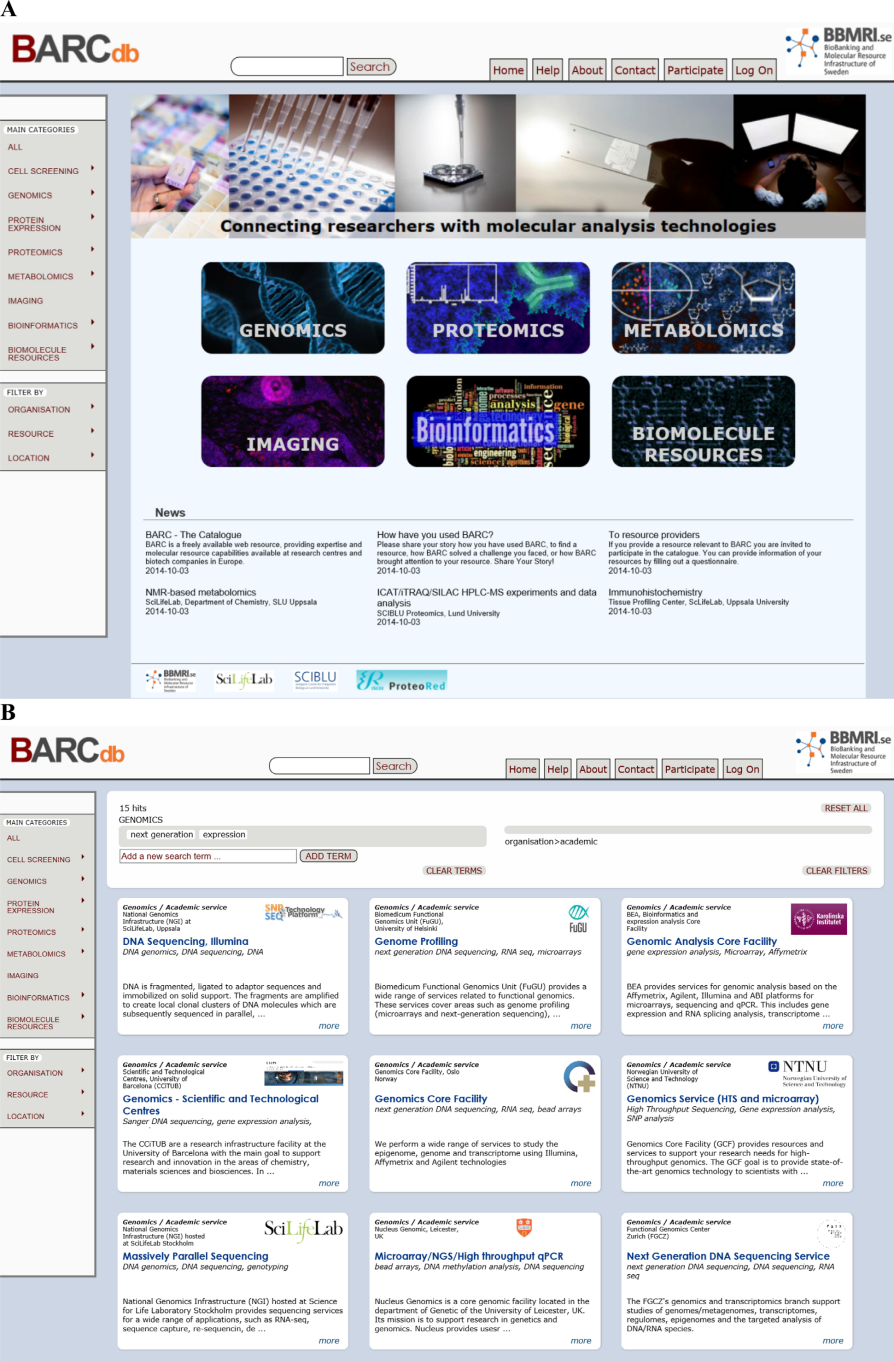
The BARCdb webpage showing (**A**) the home page and (**B**) a search result for next generation DNA sequencing resources. Individual cards function as links to more details of the analysis resource.

BARCdb presents resource and provider information in the form of ‘resource cards,’ which summarise the services available at each site and the contact details (Figure 1). Each card can be expanded with a click to provide more details. Specifications for sample requirements are included on the card, such as which types of samples can be analysed, recommended assay format, minimal sample volume, analysis throughput, and cost. The database can be searched either by free text or by categories, i.e. genomics, proteomics, metabolomics, imaging, bioinformatics and biomolecular resources. The search can also be refined by geographical location, resource type, database, product or service.

## EXAMPLES OF TECHNOLOGIES FOR BIOBANK SAMPLES

Table 1 summarise the number of resources currently included in BARCdb (October 2014). Not surprisingly perhaps, many of the providers specialise in DNA sequencing and related genome analysis. Supplementary Tables S1–S4 in the supporting material (online-only content) list all providers included. Specific examples of methods that are offered to users are:

- Array based technologies for genotyping and gene expression measurements
- Next generation DNA sequencing
- Protein analysis technologies, including both mass spectrometry and affinity-based methods
- Metabolomics by mass spectrometry and NMR
- Bioinformatics resources for analysis of data from next generation DNA sequencing, array-based gene expression, mass spectrometry, antibody-based protein biomarker analysis, etc.

**Table 1.** Present number of BARCdb analysis resources cards (October 2014)

Analysis resource type	Number
Genotyping and gene expression analysis (microarrays)	30
Next Generation DNA sequencing	27
Next Generation DNA sequencing support	8
Sanger DNA sequencing	6
High-throughput DNA extraction	5
Metabolomics	12
Proteomics, affinity-based	17
Proteomics, mass spec-based	19
Imaging	8
Other	11
Total	143

## SERVICE PROVIDER SUBMISSION PROCESS AND UPDATES

Content within BARCdb is continuously expanded and new resource providers, whether part of academic centres or companies, are invited to participate in the catalogue. An online questionnaire has been set up at http://www.barcdb.org for potential new providers. The data from the questionnaire are uploaded and a personal account is created for each provider with the possibility to update the resource information at any time. The intention is to gather information on resources for sample analysis in order to create a comprehensive Europe-wide coverage. Thus, BARCdb can be utilised by service providers to attract new customers, while for researchers it provides insights into technologies and their providers, as well as opportunities for new collaborations. The usage of the database is steadily increasing and presently the number of monthly active users is around 200 with 2000 page views.

## DISCUSSION

Biological samples are used in high-throughput techniques that allow examination of differences among individuals and over time in genomes, transcriptomes, proteomes or metabolomes and their relation, if any, to medical conditions. In addition to the significance of the data *per se*, insights derived from the analyses are also expected to drive the development of new diagnostic, prognostic and therapeutic tools, devices, reagents and drugs. Consequently, biological resources, of which the specimens stored in biobanks are a prime example, are essential raw materials for the advancement of basic life science research, biotechnology and human health. By ensuring that advanced, in some cases unique, and emerging methods are put to early and efficient use with high-quality biobank samples, scientific progress as well as commercial application by biotechnology, diagnostic and pharmaceutical industries will be promoted. Among the anticipated commercial benefits are the identification of new disease biomarkers and drug targets, together with a generally enhanced understanding of disease mechanisms.

With these broad aims in mind, we have introduced the BARCdb database as a resource for users of biobank samples searching for technologies and technology providers. BARCdb aims to fulfil an unmet need by making available, in a readily accessible form, information on molecular analysis resources and services and how and where they can be obtained, something that is not always easy to find elsewhere. The BARCdb search functions allow users to focus directly on the technologies they wish to obtain, giving researchers ‘one-stop-shop’ access to resources.

While the construction of BARCdb has been initiated within the Swedish biobanking infrastructure BBMRI.se, the resource can fulfil an important role in several EU level infrastructures within the European Strategy Forum on Research Infrastructures (ESFRI). In particular, besides the European level BBMRI, the two infrastructures EATRIS, having a focus on translational research, and ECRIN, devoted to clinical research, also depend on shared technology resources and standards, and can greatly benefit from a resource such as BARCdb.

Recently, the emerging concept of BBMRI Expert Centres has been proposed as a novel public-private partnership model stimulating transnational research collaborations (9). Expert Centres will encompass regions able to provide world-class biobanks, technology resources and medical expertise. At the request of academic scientists, major pharmaceutical firms or the diagnostic industry, such centres would undertake analyses of samples in the country of origin under internationally standardised conditions. The data would then be made available to the academic or industrial partner for further development. This can circumvent the many restrictions of exporting biological samples across borders, which otherwise makes transnational research collaboration difficult. It is intended that BARCdb will also serve as a repository of information on Expert Centres.

Another future aim for the BARCdb database is to include not only information about the methodology used by the service providers, but also whether (and if so which) standardised procedures are followed. This can be enabled by references in BARCdb to carefully documented standard operating procedures (SOPs). Such SOPs can be provided via another linked database, MolMeth (http://www.molmeth.org), which allows users to see how analyses are performed and whether data produced at different sites are generated under conditions that allow comparison of results. Increasingly, biobank related research needs to build upon patient samples collected across many different research centres in several countries. This need for ever larger studies is a direct consequence of the increasing number of molecular factors being evaluated, necessitating stronger statistical support, as well the need for replicating observations in new cohorts and across different populations. Taken together, the standardisation made possible by these databases can play an important role in supporting such international cooperation.

Samples are being collected with the intention of being used over many years, whereas technologies for analysis can be expected to improve rapidly and radically. BARCdb can help reconcile these conflicting aims by advising on foreseeable needs for sample types, thus improving the value, and accessible information content of the biobank samples being collected. Challenges in developing BARCdb further and establishing its usefulness include the wide dissemination of technology information to users of biobank samples throughout Europe, understanding user requirements and guiding the users to the most relevant analytical and pre-analytical methods out of the many possibilities available. We plan to complement the entries in the database with short reports detailing pros and cons of different techniques that may be considered for a given application. The need to keep abreast of advances in existing technologies and introduction of new technologies as they arise will also create a requirement to continuously update the database information by identifying new providers in both the academic and commercial spheres. The increased volume of data provided by DNA sequencing and other high throughput methods will also provide a constant challenge for computing and bioinformatics resources. The provision of services in this area will be of increasing importance to the biobanking community. By maintaining a wide, up-to-date coverage of technologies and providers, BARCdb can provide an important service to researchers using samples from Europe's biobanks to advance understanding, diagnostics and therapy of disease.

## SUPPLEMENTARY DATA

Supplementary Data are available at NAR Online.
